# The Outbreak of Rabies

**Published:** 1919-05-03

**Authors:** 


### THE OUTBREAK OF RABIES.

### Are More Widespread Measures Necessary?

Compared with the appalling loss of life which a variety of infections have caused in the past years of war, the worst known ravages of rabies appear somewhat insignificant, and also in this instance we have at hand an almost infallible method of treatment provided early recognition is made. Also the matter has derived some extra prominence from association in the press with questions of the Dogs' Protection Bill and Anti-Vivisection considerations, but these aspects of the case have little direct bearing on the present-day problem of again eradicating rabies from the country. We have learnt by experience in 1897 the methods to be employed, and the new situation presents no fresh complexities beyond those which allowed the re-introduction. Many letters have appeared from well-known authorities urging more thorough and drastic official action. In the main, we agree with them; on a matter of principle even though, at the time of writing, no instance of human infection with active symptoms has been reported. The fact remains, however, that already 157 cases in animals have been confirmed in the country. While they continue to arise there is always the threat that one of the most distressing forms of death may overtake some unfortunate human victim, and this alone should justify the immediate introduction of the completest preventive measures.

### The Disease on the Continent.

Rabies as an infectious disease occurs in nature epidemically among all the carnivora, but particularly the dog and the wolf. In Western Europe it most frequently affects the former, but in the wilder lands of the East, particularly in Russia, outbreaks among the wolves constitute a serious danger to man and, in fact, to most other animals. Cattle, sheep, pigs, goats, horses, and deer are all liable to infection, and the disease will occur among them in epidemic form quite readily, although the greater frequency of the canine condition has somewhat overshadowed these facts. In Northern Germany, where all dogs must be muzzled, the disease has now largely disappeared, but at one time about 700 animals were victims annually, with between seventy-five and eighty fatal cases in human beings. In France and Belgium the danger is still prevalent, and in America, according to Osler, much more frequent than is generally supposed. Before the introduction of the Pasteur treatment, although every person bitten did not contract rabies (according to Horsley about 15 per cent. only with dogs and slightly over 40 per cent. with wolves), yet the mortality in those who actually developed symptoms was over 80 per cent., and knowledge of this fact and of the very specialised procedures which must be available before diagnosis and treatment are assured, make the urgency of protective measures self-evident.

### The Mode of Infection.

The exact bacteriological nature of the virus is still unknown, but it occurs chiefly in the saliva, and to a lesser extent in other glandular secretions. It is stated that the poison reaches these glands bv the route of their nerve supply, not by the blood-stream, and certainly the relative toxicity of secretion corresponds to some extent to the richness of their innervation. In any case, the salivary glands are the richest in virus, the saliva the most potent infecting agent, and as regards its danger to man, in order of decreasing severity comes that of the wolf, cat, dog, and other animals. Of course, the bite is the common and well-known mode of transference, but the virus is so potent that any abrasion or wound licked by a rabid animal may cause infection, and cases are on record where the virus has gained entry without any obvious break in the skin surface, except the softening or roughing produced by the animal's tongue. Also experimental applications of saliva to mucous membranes such as the nose or conjunctiva are frequently followed by infection, and a moment's reflection on the habits of animals will show how readily the disease may run through packs, or be passed on to man without the injury typically pictured from a mad dog.

### The Disease in Man.

Details of the correct procedure in event of possible human infection have been widely published by official posters, pamphlets, and more recently by the current medical journals. One need only refer to a few aspects of the disease which direct the efficient carrying out of these instructions. With regard to the incubation period, Horsley finds that: (1) It is shorter in children than in adults, possibly owing to the shorter distance the virus travels before reaching the nerve centres; (2) Wounds of hands or face are usually followed by more severe and rapid onset, probably due either to the fact that these parts are mostly uncovered, whereas clothes remove saliva from teeth penetrating them, or else to the relative richness of their nerve supply; (3) The extent of the absorbing surface in deep puncture wounds and extensive lacerations reduces the latent period and increases the danger of infection. The average is about seven weeks, but variations occur between extremes of two weeks and three months, and it is only during this time that any reasonable hope of cure exists.

The importance at all times popularly attached to dog-bites cannot have escaped the notice of any surgery worker, and during an outbreak there is invaluable first-aid work, which should be done by the victim himself or his friends, especially in outlying districts, where medical assistance is not readily available. Free bleeding, cleansing, and sucking the wound should be encouraged, and thorough irrigation with water, bicarbonate, or alcohol when a recognised "antiseptic" is not at hand. The value of such immediate action is very great to the victim, but greater importance in prevention of spread attaches to the early recognition of rabid animals.

**The Outbreak of Rabies—(*continued*).**

THE ANIMAL'S CONDITION.

The prodromal stage is similar to that described for man, but in an unknown or partially domesticated animal by no means readily noticed. Also there are two distinct types of the disease, named descriptively in France *la rage furieuse* and *la rage mue*. Equal virulence of secretions occurs in both, and although in dogs the former is the more frequent, in cats, rodents, etc., the latter, or paralytic variety, is much commoner. In this case the period of excitability is so little marked that the early stages rapidly merge into the final or paralytic condition of the disease, and the animal's state may be popularly attributed to any common infection or poisoning. At a time like the present every sick beast should be treated with suspicion, and every precaution taken when in contact with it. Prompt securing, isolation, and the instructions for bringing the animal under official and expert observation should be carried out at once.

PATHOLOGY AND TREATMENT.

Of the disease itself, having gained entry at the surface, the virus spreads very slowly from the site of infection along the nerve trunks to the spinal cord, and no general symptoms develop until the centres are reached. The period of time involved varies as described above, and if meanwhile prompt action be taken, in almost every case treatment may save the patient's life. Pasteur took advantage of this period of latency, and on the principle that repeated small injections of a toxin will produce an increasing tolerance to its effects, he used injections of the emulsified spinal cords of rabbits infected with rabies. The virulence of the toxin in these cords had been reduced by drying, and, combined with quantitative graduation of the injections, it was possible to give small but increasing doses of the essential poisons of the disease. Thus before his own infection has developed the victim acquires immunity, and his protective mechanism is reinforced to meet the onslaught. The story of the original experiments, the treatment of the first case, and the remarkable results at once obtained make a fascinating and noteworthy chapter in the history of medicine. Commenced in 1885, the method was applied during ten years to 17,337 people in Paris, with an average mortality of less than ½ per cent. Nowadays even better figures are claimed, and it has been a matter of surprise that England did not long ago possess her own Pasteur Institute. Possibly this is due to anti-vivisectionist agitation, as suggested by the *Times*, but in any case we must give credit for forethought to the Board of Agriculture and the Local Government Board in providing centres at Plymouth and in London at St. Thomas's Hospital, where treatment can be given with material obtained from the Paris laboratories, injections being given daily for a period of fourteen days.

Relatively complete arrangements now exist in this country, and authorities are certainly taking more active measures on the animal side of the question. They must, however, have sympathetic co-operation and understanding from the public not only in the rural districts, but in the heart of the towns where the small but, in this instance, relatively dangerous pet dogs abound. We strongly urge that the whole country should actively assist in such measures, never treating them as an unnecessary imposition, and that full precautionary regulations should be at once enforced throughout the length and breadth of the land.

